# Replication Stress at Telomeric and Mitochondrial DNA: Common Origins and Consequences on Ageing

**DOI:** 10.3390/ijms20194959

**Published:** 2019-10-08

**Authors:** Pauline Billard, Delphine A Poncet

**Affiliations:** 1 Univ Lyon, Université Claude Bernard Lyon 1, INSERM 1052, CNRS 5286, Centre Léon Bérard, Centre de recherche en cancérologie de Lyon, 69008 Lyon, France; pma.billard@hotmail.fr; 2 Institut de Biopathologie moléculaire, Centre de Bio-Pathologie Est, Groupement hospitalier Est, Hospices Civils de Lyon, 69500 Bron, France

**Keywords:** telomere, mitochondria, replication stress, senescence, ageing, helicase, G-quadruplex, R-loop

## Abstract

Senescence is defined as a stress-induced durable cell cycle arrest. We herein revisit the origin of two of these stresses, namely mitochondrial metabolic compromise, associated with reactive oxygen species (ROS) production, and replicative senescence, activated by extreme telomere shortening. We discuss how replication stress-induced DNA damage of telomeric DNA (telDNA) and mitochondrial DNA (mtDNA) can be considered a common origin of senescence in vitro, with consequences on ageing in vivo. Unexpectedly, mtDNA and telDNA share common features indicative of a high degree of replicative stress, such as G-quadruplexes, D-loops, RNA:DNA heteroduplexes, epigenetic marks, or supercoiling. To avoid these stresses, both compartments use similar enzymatic strategies involving, for instance, endonucleases, topoisomerases, helicases, or primases. Surprisingly, many of these replication helpers are active at both telDNA and mtDNA (e.g., RNAse H1, FEN1, DNA2, RecQ helicases, Top2α, Top2β, TOP3A, DNMT1/3a/3b, SIRT1). In addition, specialized telomeric proteins, such as TERT (telomerase reverse transcriptase) and TERC (telomerase RNA component), or TIN2 (shelterin complex), shuttle from telomeres to mitochondria, and, by doing so, modulate mitochondrial metabolism and the production of ROS, in a feedback manner. Hence, mitochondria and telomeres use common weapons and cooperate to resist/prevent replication stresses, otherwise producing common consequences, namely senescence and ageing.

## 1. Introduction: Senescence and Ageing

Senescence is defined as a stress-induced durable cell cycle arrest. These stresses are of different origin, such as metabolic compromise and DNA damage, and are propagated in vivo by secretory (senescence-associated secretory phenotype (SASP)) and inflammatory pathways (for review, see [1]). Metabolic exhaustion can result from mitochondrial metabolic failure (MMF) naturally arising with age, or from oncogene overexpression during tumorigenesis. MMF also sustains a high level of reactive oxygen species (ROS) production, participating in the DNA damage senescence pathway (see below). However, this last category is mainly associated with telomere dysfunction and replicative senescence. Mechanistically, two main molecular pathways regulate senescence. The first involves p53 stabilization (for instance, following DNA damage), and p21^CIP1^ transcription. This CDKI (cyclin-dependent kinase inhibitor) in turn inhibits CDK4/6 and pRb phosphorylation, thus blocking the entry into the S-phase. The second pathway relies on an epigenetic derepression of the INK4/ARF locus, resulting in the expression of a second CDKI, p16^INK4a^, with similar consequences [2]. In vivo, the stabilization of p16^INK4a^ is concomitant to tissue degeneration during ageing in mice and in primates [2,3], and Baker et al. demonstrated that removing senescent cells delays organ degeneration and ageing using their ingenious model of INK-ATTAC mice [4,5].

In vivo, shortened telomeres and ROS are usually assumed to be the main markers of ageing and have been extensively reviewed in regard to their role in cancer initiation (for review, see [6,7]). Thus, we will hereafter focus our discussion on the contribution of telomeres and mitochondria to senescence and ageing, and present unexpected common molecular mechanisms.

## 2. The Contribution of Telomeres to Senescence and Ageing

### 2.1. Replicative Senescence or Replication Stress-Induced Senescence

Replicative senescence arises from an extreme shortening of telomeres. Indeed, in vitro, telomeres loose approximately 50–200 bp at each division (the end-replication problem). Approximately 100 mitoses are thought to be sufficient to reach the Hayflick limit and to enter replicative senescence, by activating the p53 pathway (for review, see [7]). Cells in continual renewal, such as blood cells, compensate for telomere erosion by expressing a retro-transcriptase named telomerase, the only enzyme able to polymerize telomeric sequences de novo at the extremity of telomeres. Knocking out telomerase components, such as the catalytic subunit (TERT) or the RNA template (TERC), induces several features of ageing in mice [8,9], and extremely shortened telomeres activate the DNA damage repair (DDR) pathway in vitro, thereby stabilizing p53 [10,11]. In humans, germline mutations in telomerase subunits are responsible for progeroïd syndromes, such as Dyskeratosis congenita and its aggressive form, Hoyeraal–Hreidarsson syndrome (HHS) [12]. Although these syndromes are consistent with the replicative senescence theory, the relevance of this pathway and of the Hayflick limit in physiological conditions is questionable, as telomeres are not shortened to such an extent through natural age-related erosion. Another source of telomere-driven-induced senescence could be damaged telomeres, more than extremely shortened telomeres. Indeed, telomeres adopt a specific configuration to avoid the activation of the DDR pathway, thanks to a specialized complex named the shelterin complex (or telosome; for review see [13]). This complex comprises six core proteins: telomeric repeat binding factor 1 and 2 (TRF1 and TRF2), TRF1-interacting protein 2 (TIN2), protection of telomeres protein 1 (POT1), TIN2- and POT1-interacting protein (TPP1, recently renamed ACD for adrenocortical dysplasia protein homolog), and repressor/activator protein 1 (RAP1), with specific functions in telomere protection, replication, and telomerase regulation [13]. Numerous other non-telomeric factors help in telomere maintenance, such as DDR proteins [14] or the CST complex (CTC1-STN1-TEN1), which is involved in the control of telomerase activity and in C-strand replication [15,16]. In humans, mutations in TINF2 (encoding TIN2), in ACD (encoding TPP1/ACD), and RTEL1 (regulator of telomere elongation helicase 1) (see below), are also causative of HHS, but with a drastic telomere shortening phenotype, linked to their role in telomere protection against DDR, independently of telomere length [17]. In the same way, CST depletion stops cell proliferation in vitro [16] and induces telomeropathy associated with DNA damage and senescence activation in humans [18].

Under physiological conditions, persistent telomeric damage accumulates with age, as demonstrated in the gut and liver of mice [19], and in neurons and hepatocytes of primates [3,20], irrespective of telomere length. These persistent foci are concomitant with the appearance of SAHF (senescence-associated heterochromatic foci) and p16^INK4^ stabilization [19,20], two hallmarks of senescence. At the molecular level, DNA damage at the telomere is poorly repaired [19,20] compared to other genomic sequences. Indeed, unrepaired persistent DNA damage induced by irradiation and oxidative stress mainly occurs in telomeres, whereas telomeres account for a negligible part of the initial genomic damage induced by these stresses [19,20], indicating that telomere may be considered sensors of oxidative stress. The authors also demonstrated that the expression of TERT or telomere length does not modulate persistent damage at telomeres in this situation [19].

Thus, an accumulation of unrepaired telomeric damage with age could be envisaged as the main source of telomere-driven senescence rather than extreme telomere shortening in physiological conditions. The subsequent section deals with the source of this damage. We will focus on the two main endogenous origins of telomere damage: replication defects at the telomeres and oxidative stress (linked with mitochondrial metabolism).

### 2.2. Different Sources of Replication Stress at Telomeres

**Figure 1:**
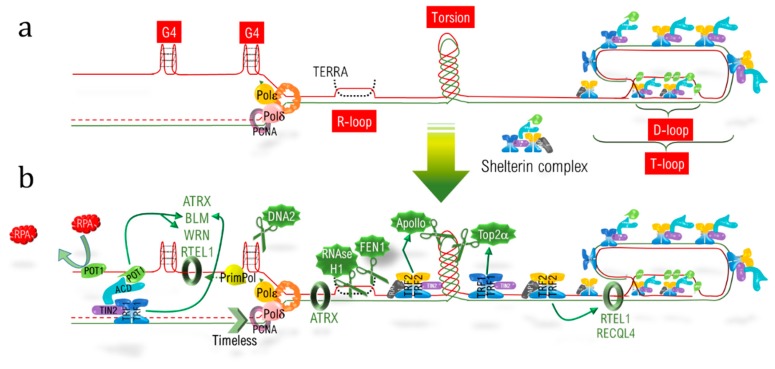
Obstacles and solutions to replicate telomeres. (**a**) Telomeric sequence, with the G-strand in solid red line and the C-strand in solid green line, is depicted. The terminal D-loop structuring the much larger T-loop is stabilized by the shelterin complex. The replisome (PCNA, Polε, Polδ…) polymerizes a new G-strand (depicted in dotted red line) and frees the parental G-strand, enabling the formation of G4. R-loops corresponding to TERRA hybridization (in dotted black lines) with the 3′-5′ strand, and torsions due to the fork progression are also shown. (**b**) Replication helpers, such as helicases (ATRX, BLM, WRN, RTEL1, and RECQL4), either helping in G4 unwinding or in D-loop unlocking, are depicted. The DNAses (Top2a, DNA2) and RNAses (RNAse H1 and FEN1) help in resolving torsions and RNA:DNA heteroduplexes, while Timeless stimulates the replisome. POT1 competes with RPA1 for binding of the single-strand and helps in G4 dissolution. The shelterin components POT1, TRF1 and TRF2 help in loading the helper-proteins (fine green arrows).

Telomeric DNA (telDNA) replication is a real challenge due to the different features of telomeres. Firstly, the nucleotidic sequence itself consists of an exanucleotidic motif (T_2_AG_3_) repeated over kilobases, with the 5′-3′ strand named the “G-strand” due to its high content in guanine. During the progression of the replication fork, the lagging strand, corresponding to the G-strand, forms G-quadruplex (G4) structures [21], which have to be resolved to allow fork progression and to complete replication (Figure 1a). Secondly, R-loops corresponding to highly stable RNA:DNA hybrids, involving the long non-coding telomeric transcript TERRA (telomeric repeat-containing RNA) [22], have to be dissociated. Thirdly, the extremity of telomeres adopts a specific loop structure, the T-loop (telomeric loop), which has to be unraveled. This loop hides the double strand end from the DNA damage sensors, and is locked by the hybridization of the 3′ single strand overhang extremity with the above 3′-5′ strand, thereby displacing the corresponding 5′-3′ strand to form a D-loop (displacement loop) structure [23,24]. Lastly, replication also has to deal with barriers encountered elsewhere in the genome, such as torsions and a condensed heterochromatic environment.

Telomeres thus possess a specialized machinery to fully achieve their replication: (i) the protein RTEL1 interacts with PCNA (proliferating cell nuclear antigen) and stimulates telomere replication [25] in parallel to Timeless, which limits fork collapse [26]; (ii) G4 at the lagging strand are unwounded by specific helicases (such as RTEL1 [27], WRN (Werner syndrome RecQ like helicase) [28], BLM (Bloom syndrome RecQ like helicase) [29], and ATRX (alpha thalassemia/mental retardation syndrome X-linked) [30]) or cleaved by the nuclease DNA2 [31]; (iii) R-loops are counteracted by ATRX [32] and eliminated by the RNA:DNA nuclease RNAse H1 [33] or by the flap endonuclease FEN1 [34]; (iv) supercoiling is resolved by nuclease such as Top2α and β [35,36] or Apollo (hSNM1B) [37]; and (v) RTEL1 and RECQL4 (RecQ helicase-like 4) help to dissociate the terminal D-loop structure (Figure 1b) [27,38,39].

The recruitment of these factors is orchestrated by the shelterin complex. On the one hand, TRF2 triggers replication by recruiting the ORC (origin replication complex) [40], alleviates the topological stress by recruiting Apollo [37], and allows T-loop dismantlement together with RTEL1 [38] and RECQL4 [39]. On the other hand, TRF1 helps fork progression by recruiting Timeless [26], favors the R-loop dissociation by interacting with BLM [29], and relocates POT1 at the G-strand [29,41]. Hence, POT1 stimulates WRN and BLM helicase activity to linearize G4 [28] and limits their formation by direct binding [42].

The silencing of these telomere replication-helper factors (such as TRF1 [29,43,44], TIN2 [29], RTEL1 [25,44], RECQL4 [39], BLM [29,44], DNA2 [31], FEN1 [34], Timeless [26], or TOP2A [45]) results in replication stress characterized by multiple telomeric signals and sister-telomere associations during metaphase, a phenotype defined as fragile telomere. This process is concomitant to the induction of senescence [25,43,46]. In humans, germline mutations in the replication helpers TRF1, POT1, and ACD are causative of pulmonary fibrosis, a pathology resulting from an excess of senescence, and mutations in WRN and RECQ4L result in accelerated ageing syndromes associated with telomere dysfunctions (for reviews, see [12,47,48]). Moreover, telomeres with unrepaired damage, in a context of defective DNA repair, can be fused together and initiate cycles of anaphase bridge/break and fusion. This process is a well-known motor for cancer development, a disease associated with ageing, and has been especially well documented in the context of telomere replication-helper deficiency (for review, see [7]).

Collectively, DNA damage resulting from failure of replication at telomeres induces senescence in vitro, irrespective of the size of telomeres, and is associated with ageing in vivo.

### 2.3. Oxidative Stress at Telomeres: Origin and Regulation

Another endogenous source of telomeric damage is ROS. Among the different subcellular compartments, mitochondria are the main source of ROS, with approximately 1–3% of the total molecular oxygen consumption transformed into superoxide anions [49]. Ageing in primary cells, whether or not they express telomerase, such as T CD8+ lymphocyte [50] or fibroblasts [51,52], is associated with a gradual increase in ROS production due to progressive mitochondrial failure, and is concomitant to telomere shortening. The neutralization of ROS does not restore the mitochondrial function but inhibits telomere shortening [50,52], establishing ROS as the source of telomere shortening. In addition, human syndromes associated with chronic ROS hyperproduction, such as mitochondriopathies, effectively show a decrease in telomere length [53,54].

At the molecular level, the GGG telomeric repeats are particularly sensitive to ROS [55], which produce stretches of 8-oxoguanine, especially difficult to repair. Coupled with inefficient telomere repair, these ROS-induced lesions produce single and double-strand breaks, and/or generate replicative stress, ultimately resulting in telomere shortening [56,57]. The presence of unrepaired single or tandem 8-oxoguanine drastically inhibits the binding of TRF1 and TRF2 [58], and impairs the recruitment of telomerase, when localized in the 3′ overhang [59], thereby contributing to telomere deprotection and shortening. As an example, oxidative stress accelerates telomere shortening—when combined with a telomerase inhibitor—only in cells deficient for oxidative damage repair, such as fanconi anemia-derived cell lines [60].

In response, cells have developed strategies to counteract the deleterious telomeric consequences of acute ROS production. The 8-oxoguanine DNA glycolylase OGG1, involved in 8-oxoguanine repair, for instance, plays an important role at telomeres. Its invalidation induces a chronic replication stress and an accelerated telomere loss [61]. ROS also control numerous pathways via the oxidation of specific cysteine residues in enzymes, thereby modulating the conformation and activity of its targeted enzymes [62]. Among them, peroxyredoxin 1 (PRDX1; activated by acute and chronic oxidative stresses) localizes at the telomeres and reduces the presence of oxidized bases by recruiting MTH1 (7,8-dihydro-8-oxoguanine triphosphatase) [59,63]. MTH1 is involved in oxidized dGTP clearing, avoiding its incorporation into DNA. The inactivation of MTH1 and PRDX1 thus results in terminal 8-oxoguanine insertion that further blocks elongation and results in telomere shortening [59,63].

Strikingly, ROS (and other metabolic stresses) also induce the relocation of TERT to mitochondria, as observed (i) in primary neurons after oxidative stress [64]; (ii) in neurons exposed to the tau protein [64]; (iii) in Purkinje neurons subjected to excitotoxicity [65]; and (iv) in cancer cell lines treated with a G4 ligand [66]. In humans, during the course of viral-induced hepatic cancer, primary hepatocytes support a gradual increase in ROS production, telomere shortening, and re-expression of TERT, which localizes in mitochondria. This results in a lower level of 8-oxoguanine in mtDNA than in nuclear DNA [67]. In the parasite Leishmania major, the relocation of TERT (LmTERT) to mitochondria in the case of oxidative stress protects cells from cell death [68]. Mice with dietary restriction-induced mTOR (mechanistic target of rapamycin kinase) inhibition or treated by rapamycin (mTOR inhibitor) show an increased localization of TERT at mitochondria in the brain [69], associated with a decrease in ROS and an improvement in the learning and memorizing capacities of mice. Inversely, knocking out mTERT is responsible for mitochondrial respiratory defects in primary cardiomyocytes well before telomere shortening [70].

Regarding the subcellular signalization implicated, in vitro, oxidative stress or mTOR inhibition induces the nuclear export of TERT, by its phosphorylation at the Y707 position by Src [69,71], and its relocation to the mitochondrial matrix [70], owing to its mitochondrial-targeting signal (MTS) [72,73]. Mitochondrial TERT increases the inner membrane potential, as well as the mtDNA copy number, and decreases ROS production [70,74], with a protective effect on mtDNA [67,70,75] and nDNA against oxidative damage [70,74]. This also impinges on the elimination of dysfunctional mitochondria by mitophagy [76]. The detailed molecular mechanisms underlying this process remain unclear. TERT (and LmTERT in Leishmania major) can directly bind different regions of the mitochondrial DNA (mtDNA) [70,73] (and kinetoplast) [68] and interact with 14 tRNAs in the nucleoid [68]. TERT also acts as a retro-transcriptase in vitro [73] and displays a de novo [77] RdRP (RNA-dependent RNA polymerase) activity involved in RNA interference in the nucleus [78]. These activities may, in fine, regulate the concentration of mitochondria-encoded tRNA and influence the replication of mtDNA (in the RNA incorporation throughout the lagging strand mode (RITOLS), see below). Thus, TERT controls a feedback loop regulating ROS production and mitochondrial healthiness, stimulating mtDNA maintenance, with a probable implication on its anti-ageing effects [79,80]. Other actors of telomere maintenance are also present in mitochondria, such as TERC [81] or the shelterin component TIN2 [82] (with an opposite effect on ROS than observed with TERT). Other telomeric factors also present an important extra-nuclear fraction and modulate the mitochondrial metabolism, such as ACD/TPP1, RAP1, POT1 [46,83], or a variant of dyskerin [84,85].

To conclude, mitochondria-produced ROS are direct inducers of DNA damage at telDNA, which results in accelerated telomere shortening and senescence, with implications for ageing. Cells have developed defense mechanisms to control ROS-induced damage at telomeres, and to reduce mitochondrial production of ROS in a feedback manner, a process involving the shuttling of telomeric proteins into mitochondria. However, mitochondria-generated ROS are not the only contributors of mitochondrial to cell entry into senescence. We will now discuss how dysregulation of mtDNA replication, like telDNA, can be a driver of senescence.

## 3. The Origin of the Mitochondrial Metabolic Compromise during Ageing

Destructuration of cristae, alteration of mitochondrial morphology, and dysregulation of mitochondrial metabolism are increasingly considered senescence markers (for review, see [86]), placing mitochondrial hormesis as a senescence gatekeeper.

Regarding the molecular events associated with mitochondrial dyshormesis, electron chain transport (ECT) deficiency or inhibition induces the accumulation of its substrates (NADH, H+ and FADH2) responsible for a retrograde inhibition of the mitochondrial metabolic pathways (Krebs cycle, pyruvate dehydrogenase, beta-oxydation of fatty-acids) and results in global MMF. This situation is also associated with a burst in ROS, due to leaky electrons from the ECT, which directly react with molecular oxygen to produce superoxide radicals [49]. The following cell signaling pathway thus linking MMF to senescence induction via p53/p21 or pRb/p16 pathways is not straightforward and involves ROS (and the telomeric hub, as detailed above), and also ATP or NAD+ levels [87], as detailed in [88].

In the present review, we will focus on the origin of ECT dysfunction and MMF during ageing. The commonly admitted theory of ageing relies on a progressive increase in mitochondrial ROS production. Locally-produced ROS are supposed to mainly target mtDNA in close proximity. As mtDNA encodes for ECT components, a vicious circle, increasing ROS production and mtDNA alterations, is thus expected to occur. The main DNA lesion produced by ROS is 8-oxoguanine. If this lesion remains unrepaired by OGG1 coupled with base excision repair (BER) in mitochondria, 8-oxoguanine can be paired with adenine, and thus generates a G:C to T:A transversion mutation, making this transversion characteristic of oxidative damage. However, invalidation of OGG1 and MUTYH (mutY DNA glycosylase) (involved in base excision repair after oxidative stress) in mice, either by knocking out [89] or by knocking in [90] a version unable to target mitochondria, does not increase this mtDNA oxidative mutation, even after inbreeding with a superoxide dismutase SOD2-deficient mouse model, while ROS are clearly boosted [90]. Consistently, in humans, only 4% of the mutations accumulated with age are related to oxidative stress [91,92], whereas the majority of mutations result from replication errors [92,93]. One could thus, as for telomeres, speculate that replication stress is an initiator of MMF associated with age.

Indeed, in vivo studies performed on transgenic mice with an error-prone version of the mitochondrial DNA polymerase gamma (polγ) show an increase in mtDNA mutations, in ROS production (resulting from ECT dysfunction) [94], and in senescence [87]. Mice also demonstrate a progeroïd phenotype with clinical signs of ageing, such as alopecia and graying, kyphosis, osteoporosis, sarcopenia, anemia, cardiac hypertrophy, and a reduced lifespan [94,95]. Another clue as to replication defects in ageing is the recurrent deletion of approximately 5 kb (nucleotides 8.483–13.459) [96] accumulating with age in humans in the cortex, muscle, liver, and testes [97,98]. This deletion encompasses 12 genes, including 5 tRNA, ultimately preventing the synthesis of the 12 mtDNA-encoded proteins. The transfer of deleted mtDNA copies in fertilized ovocytes results in accelerated ageing in mice [99,100]. This 5 kb-long fragment is deliminated by two identical sequences of 13 bp, with only one repeat remaining in deleted mtDNA. This deletion is believed to occur during the strand displacement mode (SDM) of mtDNA replication and has recently been attributed to a copy-choice recombination process [101]. A mutated version of polγ corresponding to the pathological version reported in autosomal dominant progressive external ophthalmoplegia (adPEO), increases the rate of appearance of this 5-kb deleted mtDNA [101]. At the clinical level, mitochondriopathy caused by polγ mutations shows clinical signs of ageing, such as cataracts, cardiomyopathy, or premature menopause, in addition to muscular and neuronal degeneration [102].

Hence, mtDNA replication errors accumulating during the lifespan are a driving force of MMF and ageing, in a manner reminiscent of that observed for telomere replication defects.

## 4. mtDNA and telDNA Maintenance: The Same Causes Produce the Same Effects

The origin of this unexpected common mechanism of replication stress fueling senescence and ageing may be a common features of DNA organization at telomeres and mtDNA. Indeed, mtDNA and telDNA share common particularities, such as a D-loops, epigenetic constraints, G4, RNA:DNA hybrids, and have to deal with torsion, rendering their replication highly sensitive.

### 4.1. D-loops

As for the D-loop structuring the T-loop at telomeres, mitochondria can also present a D-loop structure. This D-loop is part of the largest non-coding region (NCR) of mitochondria encompassing the heavy strand origin of replication (OH), as well as the light and the heavy strand promoter (HSP, LSP) (for review, see [103,104]). A regulatory mechanism enables the transcription, at the HSP, of a 100 base RNA that serves to prime the 7S DNA synthesis by polγ. The 7S DNA remains associated with the parental strand, thus displacing the H strand (heavy strand) in a D-loop manner. The tight regulation of 7S DNA synthesis implicates numerous factors. The D-loop plays an essential function in regulating the balance between replication and transcription and can regulate the pool of mitochondrial nucleotides [103,104]. During replication, D-loops have to be unlocked to allow the replisome to proceed. At telomeres, RECQL4 (a RecQ helicase) interacts with shelterin components and with the helicase WRN to help in replicating damaged telomeres and to unwind the D-loop [39]. In mitochondria RECQL4 is also essential for mtDNA replication in connection with the helicase Twinkle, and is helpful for replicating mtDNA after oxidative DNA damage (Table 1) [105,106]. Germline mutations in RECQL4 recapitulate a progeroïd syndrome resembling Dyskeratosis congenita (for review, see [48]).

RAD51/XRCC3 have been observed in the D-loop region [107], such as holiday junctions [108,109], suggesting the existence of homologous recombination at this site. This hypothesis has been confirmed by the exchange between paternal and maternal material in the D-loop sequence, in a peculiar pathological situation [110]. RAD51/XRCC3 are also recruited elsewhere in mtDNA after DNA damage and help to cope with replication stress, ensuring mtDNA integrity [107]. Homologous recombination also operates at telDNA, enabling the copy of telomeric sequences from another telomere, in the ALT (alternative lengthening of telomeres) process. It also involves a RAD51-dependent pathway and is also initiated by replication stress (Table 1) (for review, see [111]).

### 4.2. Epigenetic Regulation

An important epigenetic marker on nuclear DNA (nDNA) consists of the methylation of CpG dinucleotide. When clustered on the gene promoter, CpG island methylation represses gene transcription, while methylated interspersed CpG is associated with heterochromatin features. Regarding mtDNA, CpG does not seem to be the major target of methylation. However, the D-loop region is the most enriched in this marker [123] and its methylation involves the DNA methyltransferases (DNMT) DNMT1 [124,125] 3a and 3b (Table 1) [126,127]. Knocking down these DNMT induces senescence in vitro, and their expression is naturally downregulated in senescent cells [127]. In humans, dysregulation in the mitochondrial D-loop methylation has been reported in age-related neurodegenerative disorders [128]. One of the characteristics of telomeric chromatin is the high level of methylation of the subtelomeric sequences (CpG dinucleotide are absent from the T_2_AG_3_ telomeric motif). DNMT1 and DNMT3a and 3b also play crucial roles at telomeres, as their invalidation leads to telomere damage, elongation, and genomic instability [129]. The natural extinction of DNMT1 enables telomere elongation and reprogramming at the embryonic two-cell stage [130]. Regarding the demethylation of methylated CpG (mCpG), a first step of oxidation producing the 5-hydroxymethylcytosine (5hmC) is catalyzed by oxygenase of the ten-eleven-translocation (TET) family. Evidence of 5hmC has been reported in mtDNA [131], and TET1 and TET2 have been reported in mitochondria [132]. Demethylation of the D-loop could be involved in the initiation of transcription [131], and its role in mtDNA replication has so far not been addressed. In the nucleus, knocking out TET1 and TET2 increases methylation of the subtelomeric region and induces telomere shortening in mouse embryonic stem cells [133]. Conversely, a low methylation of the subtelomeric promoter of TERRA induces its transcription and is associated with telomere elongation in placenta [134]. In humans, in age-associated diseases, subtelomeric methylation and telomere length are dysregulated [135]. Thus DNMT1, 3a, 3b, and TET1,2 regulate mtDNA and telDNA integrity, and the transcription and dysregulation of CpG methylation in both types of DNA is reported in age-related diseases.

Considering chromatin, mtDNA is compacted by the protein TFAM (transcription factor A, mitochondrial) into a mitochondrial chromosoma. In humans, pathologies generated by germline mutations in TFAM demonstrate MMF and clinical signs of ageing (cardiopathy, myopathy, diabetes, neurodegeneration) (for review, see [136]). As for histone into chromatin, the acetylation of TFAM reduces its interaction with mtDNA [137], and deacetylases of the sirtuin family, such as SIRT1 and SIRT3, reduce TFAM acetylation, increase mitochondrial biogenesis [138,139], and inhibit senescence [87]. At telomeres, a variant of histone 3, H3.3, increases with ageing in different tissues [140], and plays a role in longevity [141]. Telomeres are also characterized by repressive histone marks, such as H3K9^me3^ and H4K20^me3^. At telomeres, SIRT6 is responsible for H3K9 deacetylation, its invalidation leads to replication defects, unrepaired DNA damage, and generates in knock out mice an accelerated ageing phenotype (for review, see [142]). SIRT1 also play a role in telomere compaction and integrity. Its gain in function, in mouse models, attenuates telomere shortening during ageing, while its invalidation leads to a reduction in H3K9^me3^ and fragile telomeres [143]. In humans, a polymorphism in SIRT1 has even been associated with longevity and long telomeres [144]. Overall, the acetylation of TFAM and H3K9 in mtDNA and telDNA, respectively, relies on sirtuins and is involved in senescence and ageing.

### 4.3. G-Quadruplex

A striking common point between mtDNA and telDNA is the presence of a strand enriched with guanine, named the heavy strand (H strand) in mtDNA and G-strand in telDNA. These DNA are able to adopt G4 conformations while released as a single strand during replication.

**Figure 2:**
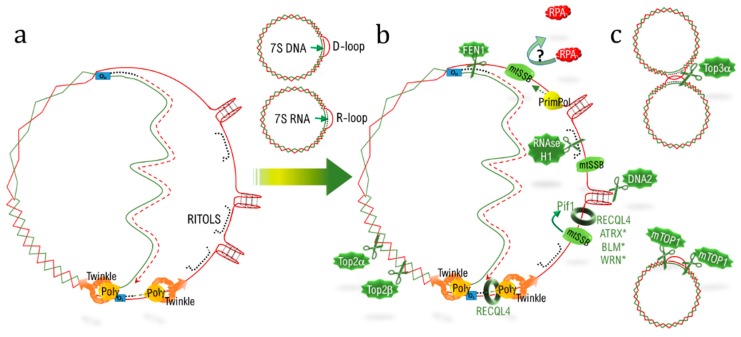
Obstacles and solutions during mtDNA replication. (**a**) The mitochondrial replisome (Polγ, Twinkle) replicates the leading strand (dotted red line) complementarily to the light strand (green line) until it frees the O_L_ enabling the replication of the lagging strand (doted green line). The freed lagging strand (in red) forms the G4 and R-loop (in agreement with the RITOLS replication mode, with RNA depicted in dotted dark lines). (**b**) Numerous factors allow for an accurate replication, by cleaving (DNA2) or unwinding G4 (Pif1, RECQL4, ATRX, BLM, WRN); by suppressing RNA:DNA hybrids (RNAse H1, FEN1), or by alleviating torsions (TOP2a, TOP2b, mTOP1, TOP3a). mtSSB stabilizes the single-stranded H-strand and stimulates Pif1 helicase activity. (**c**) Top3a decatenates the two copies of mtDNA resulting from the replication and mTOP1 reduces supercoiling surrounding the D-loop.

Two modes of replication exist in mtDNA (as a function of metabolic environment [108] or mitochondrial transcription level [145]): the SDM (see above) and the strand-coupled model (SCM) (for complete reviews on the subject, see [104,146]). The two origins of replication O_L_ and O_H_ are separated by approximately two thirds of the total mtDNA length. During the SDM, polγ, alongside the helicase Twinkle, starts to replicate the L strand, and once the OH is freed by the replisome, the H strand can be replicated. During the lapse of time enabling polγ to reach the O_H_, the H strain is thus in a single-stranded conformation and can produce G4. Over 80 sites are predicted to form G4 in mtDNA and are real obstacles for lagging strand replication, resulting in mutation hotspots in mtDNA [147]. G4 are enriched in mtDNA compared to nDNA, explaining the particular mitochondrial toxicity of the G4-ligand [148]. As for telomere replication, mitochondria have developed strategies to counteract G4 during replication (Figure 2). First, the mitochondrial protein mtSSB (mitochondrial single strand binding protein) covers and stabilizes the single strand [149], similar to POT1 [42,150,151], which inhibits G4 formation (Table 1). At telomeres, POT1 also competes with RPA (replication protein A) binding, thereby inhibiting DDR activation [150,152,153], a phenomenon that is facilitated by TERRA, which removes RPA from single-stranded telDNA [152]. Long-non coding mtRNA (mt-lncRNA) stays associated with the single-stranded mtDNA H-strand in the RITOLS models (ribonucleotide incorporation throughout the lagging strand) of replication and RPA3, one of the subunits of RPA which has been involved in mitochondrial repair via functional screens [122]. One could envisage the existence of an interplay between mtSSB and mt-lncRNA at single-stranded H strand to avoid DDR engagement during mtDNA replication.

Helicases also play a crucial role in the nucleus, where they help with G4 unwinding during replication, such as Pif1 [154] and RECQL4 (Table 1) [155]. Recently, a functional screen demonstrated an implication of three additional helicases, BLM, WRN (of the RecQ family), and ATRX—all three also acting at telomeres (see above)—in mtDNA maintenance [122]. In addition, human syndromes due to BLM or WRN deficiency are associated with MMF and ageing [156]. Moreover, a cooperation between the single-strand binding proteins and helicases to help in replicating G4 is observed in both mtDNA and telDNA. Indeed, mtSSB (single stranded DNA binding protein 1) and Pif1 can cooperate to help polγ in mtDNA replication at G4 sites [157], similarly, POT1 stimulates WRN and BLM to unwind G4 at telomeres [28]. For unsolved G4 structure, cleavage is also a solution, as previously discussed for DNA2 at telomeres, which ensures replication and genomic stability [31]. DNA2 is also located in mitochondria, where it accumulates at replication fork arrest and helps in mtDNA repair [158].

Lastly, another strategy involves re-priming the replication after G4-induced fork collapse. The specialized DNA polymerase PrimPOL fulfils this function at G4 sequences in mitochondria and in the nucleus (see below). Hence, telDNA and mtDNA are prone to G4 formation and share the same arsenal of single-strand binding proteins, nucleases, helicases, and primases to ensure adequate replication.

### 4.4. RNA:DNA Hybrids

There are two main origins for RNA:DNA heteroduplexes in mitochondria. First, in the RITOLS mode of replication, the lagging strand is supposed to be stabilized by its hybridization to long processed mitochondrial transcripts, before its replication [159]. At telomeres, the long telomeric transcript TERRA is also associated with telDNA in a RNA:DNA heteroduplex fashion, displacing the G-strand and forming a R-loop. The second heteroduplex of importance in mitochondria is the R-loop—this loop involves a 7S RNA instead of the 7S DNA, turning the D-loop into a R-loop, with specific functions in mtDNA segregation [103]. The RNAse H1 targets RNA into a heteroduplex; two isoforms are translated from different start sites, one is nuclear and the other mitochondrial (Table 1). A pathological excess of activity of the RNAse H1 has been identified in aberrant mtDNA segregation due to the elimination of the 7S RNA in the R-loop [160]. Moreover, specific deletion of the mitochondrial isoform induces a rapid loss of mtDNA and cell death [161], which could also be linked with long transcript removal in the RITOLS model. The nuclear isoform of RNAse H1 is essential for R-loop dissociation at telomeres and is crucial to achieve telomere replication [33]. As for RNAse H1, the flap endonuclease FEN1 is targeted to mitochondria via an alternative translation start and plays a role in RNA degradation in the R-loop of the NCR [162]; its overexpression abolishes mtDNA replication, probably due to the removal of the RNA primer necessary to prime polγ at the NCR. At telomeres, FEN1 resolves the R-loop involving TERRA and allows replication and stability [163].

Obstacles such as R-loops or G4, if unresolved, induce fork collapse. A specific DNA primase polymerase PrimPol is able to re-prime replication at sites of unrepaired DNA-damage in the nucleus and in mitochondria [164]. Its activity is of utmost importance to manage R-loop [165]- and G4 [166]-induced fork collapse in the nucleus. Its invalidation impairs mtDNA replication [164], probably in association with G4 and R-loop accumulation, but its specific function at telomeres, inside the nucleus, has not yet been studied.

To summarize, RNAse H1, FEN1, and PrimPol help to cope with heteroduplexes at mtDNA and telDNA, enabling replication and genome maintenance.

### 4.5. Torsion and Supercoiling

Mitochondria can adopt different conformations according to their activity. The type II topoisomerase Top2α and Top2β are important for regulating these conformational transitions; their inhibition by ciprofloxacin induces an accumulation of positively supercoiled mtDNA, impeding mtDNA replication and transcription and finally resulting in cell toxicity [167]. In the nucleus, Top2α and Top2β also help in telomere replication and segregation [37]. Top2α, in combination with TRF1, helps in telomere disentangling, and its invalidation results in the formation of ultrafine bridges during mitosis [45]. Its inhibition in cells with long telomeres induces replication defects and a drastic reduction in telomere length, probably in association with excessive positive supercoiling at the head of the replication fork during replication [36].

Once replicated, the two mtDNA copies form hemicatenanes, which are decatenated by the type I topoisomerase Top3α [168]. Biallelic mutation of TOP3A is reported in a patient suffering from chronic progressive external ophthalmoplegia (CPEO), characterized by muscle-restricted mtDNA deletions [168]. At telomeres, the dissolvasome, or BTR (BLM-TOP3A-RMI) complex, enable the dissociation of recombination intermediates without exchange, making the ALT process a conservative DNA replication mode [169]. In humans, mutations in BLM or in BTR components produce severe syndromes with growth retardation and increase the risk of cancer [170]. In vitro, cancers are related to genomic instability and sister-chromatid exchange at telomeres, while metabolic failure is related to the mitochondrial function of Top3α [170].

A strictly mitochondrial class I topoisomerase, mTOP1 helps in relaxing supercoiled mtDNA by cutting two specific palindromic sequences bordering the D-loop. Its depletion leads to a rapid loss of mtDNA, independently of mitochondria mass decrease [171]. Its nuclear homologue TOP1, in yeast, is associated with proper replication of G4-enriched DNA sequences and is essential for genomic stability [172], but its specific function at telomeres has not been investigated.

## 5. Concluding Remarks and Future Perspectives

Because one of the main differences between chromosomes and mitochondrial chromosoma is the absence of telomeres, it is surprising to see how telDNA and mtDNA share common properties and maintenance mechanisms. One of the main outcomes is a high level of replicative stress, with a common consequence on senescence induction in vitro and on ageing in vivo. This stress can also be considered the origin of the two conventional ageing-associated markers, namely ROS and telomere shortening.

Moreover, in sus of the 1158 mitochondrial proteins listed in the mitocarta database (https://www.broadinstitute.org), additional proteins without a conventional N-terminal MTS can be located in mitochondria, likely by an alternative translation initiation [154,161]. The pool of mitochondrial isoforms is probably underestimated, due to the absence of the stimulus responsible for their specific targeting of mitochondria (for review, see [173]). Thus, Wisnovsky et al. recently identified, via a functional screen, at least 31 genes involved in nDNA maintenance (replication and repair) acting in mitochondria [122]. We can thus postulate that the list of common actors of mtDNA and telDNA maintenance is longer than those summarized in this review.

## Figures and Tables

**Table 1:** Listing of the common or equivalent proteins influencing replication at both telomere (telDNA) and mitochondrial DNA (mtDNA) (in mammals).

Mechanisms	Features/Actors	*mtDNA*	*telDNA*
***Structuration***	**D-Loop**	D-loop (NCR)	D-loop (T-loop)
	**G rich sequences**	H strand, G4 +++	G strand, G4 +++
	**Curvature**	TFAM [112]	TRF2 [113]
	**RNA:DNA hybrid**	R-loop (7SRNA), RITOLS §	R-loop(TERRA) §
	**Specialized telomeric factors**	TERT, TERC, TIN2 §	Telomerase complex, shelterin complex §
***Chromatin organization***	**Specific proteins**	TFAM §	H3.3 §
	**Epigenetic modulators**	DNMT1, 3b §	DNMT1/3A/3B §
		TET1, TET2 § SIRT1/3/4/5 §	TET1, TET 2, TET3 § SIRT1, SIRT6 §
	**Structuration**	SMC6 *	SMC6 [114]
***Replication***	**Polymerase**	POLG §, POLQ *	POLD, POLE §, POLQ [115]
	**(Translesional)**	REV3L *	Pol η [116]
	**ssDNA binding proteins**	mtSSB §, RPA3 *	POT1, RPA §
	**RNAse**	RNASe H1 §	RNAse H1 §
	**Torsion/supercoiling**	TOP1mt, TOP2α, TOP2β §	TOP1 #, TOP2α, TOP2β §
	**G4 unwinding,** **G4 clivage**	Pif1, ATRX *, WRN *, BLM *, DNA2	Pif1#, ATRX, WRN, BLM, DNA2
	**Primase**	PrimPol	PrimPol #
	**D-loop dissociation**	RECQL4/Twinkle #	RECQL4/WRN
***Homologous recombination***	**Homology search/maturation**	ctIP [117], RAD51 [107], MRE11 [117] XRCC3 [107]	ctIP [118], RAD51, MRE11 XRCC3 [119]
	**Endonucleases**	EXO1 *, GEN1 *	EXO1 [120], GEN1 [121]
		MUS81 *	MUS81 [121]
	**Flap-exonuclease**	FEN1, DNA2 §	FEN1, DNA2 §
	**dJH resolutions**	TOP3A §	TOP3A §

* identified by functional screen without further confirmation [122], # Localization confirmed, but function not fully addressed, § the corresponding references are mentioned in the text.

## Abbreviations

5hmC	5-hydroxymethylcytosine
adPEO	autosomal dominant progressive external ophthalmoplegia
ALT	alternative lengthening of telomeres
CDKI	CDKI cyclin-dependent kinase inhibitor
DDR	DNA damage repair
D-loop	displacement loop
DNMT	DNA methyltransferases
ECT	electron chain transport
G4	G-quadruplex
HHS	Hoyeraal-Hreidarsson syndrome
HSP	heavy strand promoter
LSP	light strand promoter
MMF	mitochondrial metabolic failure
mtDNA	mitochondrial DNA
mt-lncRNA	long-non coding mtRNA
mt-lncRNA	long-non coding mtRNA
MTS	mitochondrial targeting signal
nDNA	nuclear DNA
O_H_	heavy strand origin of replication
O_L_	ligth strand origin of replication
ORC	origin replication complex
RdRP	RNA-dependent RNA Polymerase
RITOLS	ribonucleotide incorporation throughout the lagging strand
ROS	reactive oxygen species
SASP	senescence-associated secretory phenotype
telDNA	telomeric DNA
TET	ten-eleven-translocation
T-loop	telomeric loop
